# Point-of-Use QA in Digital Pathology Slides

**DOI:** 10.1155/2014/590813

**Published:** 2014-12-30

**Authors:** David Brettle, Darren Treanor, Craig Revie, Mike Shires

**Affiliations:** ^1^Leeds Teaching Hospitals NHS Trust, Leeds LS14 6UH, UK; ^2^FFEI, Hertfordshire HP2 7DF, UK; ^3^University of Leeds, Leeds LS2 9JT, UK

## Background

In digital histopathology traditionally prepared and stained slides are scanned in a dedicated scanner to produce extremely high resolution images. The resultant image fidelity is affected by many variables including the staining processes, scanner design/setup, and ultimately the image display. Little or no routine quality control is applied at any of these stages and as a result widely varying images can be produced for the same sample.

## Method

It is proposed that an intraslide test tool would allow each variable in the imaging chain to be quantified. This will allow routine quality control monitoring as well as the ability to normalize or correct the image. A Point-of-Use QA (POUQA) test tool is proposed made of two key zones that can be applied to every slide.

*Zone 1*. Fixed color patches allow accurate positioning of the resultant image in colour space. The patches can be either for a wide gamut of color space or more localized depending on the stain.

*Zone 2*. It is comprised of a suitable substrate that will take up the stain proportionally to the clinical sample. This will allow quantification of the variability in staining and provide a reference for slide fading.

Other zones can be added as required, for example, for white balance or resolution measurement.

## Results

This presentation will include initial stage proof of concept results of an intraslide QA tool using H&E stain and demonstrate how slide variability can be normalized.

## Conclusion

A digital pathology POUQA test tool has been developed that allows both color and stain assessment and introduces image assurance into potentially every digital pathology slide.

